# Case Report: Isolated male epispadias in adult: a rare case report

**DOI:** 10.3389/fruro.2026.1811127

**Published:** 2026-04-23

**Authors:** Rifki Adhi Nofrian, Joko Pitoyo, Safendra Siregar, Albert Ciam, Muhammad Fadel Yudawa

**Affiliations:** 1 Urology Department, Hasan Sadikin Academic Medical Center, Universitas Padjajaran, Bandung, Indonesia; 2 Department of Surgery, Urology Sub-Division, Faculty of Medicine, Riau University, Pekanbaru, Indonesia

**Keywords:** adult epispadias, epispadias repair, isolated epispadias, modified Cantwell-Ransley, reconstructive urology

## Abstract

**Introduction:**

Isolated male epispadias is a rare congenital anomaly characterized by dorsal urethral displacement, typically corrected during infancy. Cases presenting for the first time in adulthood are exceedingly rare and pose unique challenges regarding surgical reconstruction and psychosexual well-being.

**Case presentation:**

A 35-year-old continent male presented with a dorsal midshaft urethral meatus and associated chordee, seeking correction prior to marriage. The patient underwent a modified Cantwell-Ransley procedure, involving degloving, chordee excision, and ventral transposition of the urethral plate. Postoperative recovery was noted for transient minor glans necrosis, which healed spontaneously. At the three-month follow-up, the patient achieved a functional result with a peak flow rate () of 16 ml/s and an IIEF-5 score of 22/25.

**Conclusions:**

Surgical correction of isolated epispadias in adulthood is feasible and offers significant benefits. The modified Cantwell-Ransley technique is a reliable approach for restoring anatomical integrity and sexual function.

## Introduction

Epispadias is a rare congenital anomaly that occurs in approximately 1 in 117,000 live births and is characterized by dorsal displacement of the urethral meatus. This condition is frequently associated with varying degree1756s of penile deformity and urinary incontinence (1). Clinically, epispadias may present as an isolated anomaly or as part of the bladder exstrophy epispadias complex (BEEC). Isolated male epispadias accounts for less than 10% of all cases and does not involve bladder or pelvic abnormalities (2). Adult presentations are particularly uncommon, with outpatient treatment rates reported as low as 0.6 per 100,000 annually (2, 3). The severity of epispadias is determined by the degree of failure in urethral plate tubularization and is classified into three subtypes: glanular, penile, and complete epispadias (2).

These anomalies are frequently associated with an excess of ventral preputial skin and a deficiency of dorsal penile skin. Currently, there is no standardized consensus regarding the surgical management of isolated epispadias. Various techniques, such as the Cantwell-Ransley, Mitchell, and Kelly procedures, have been described, each offering distinct perspectives and outcomes (4). Although surgical correction is typically performed within the first year of life, some cases are delayed until adolescence or adulthood. Delayed presentations are associated with significant psychosocial and psychosexual challenges, as well as increased surgical complexity (5). This study presents a case of adult isolated male epispadias and examines surgical repair techniques and outcomes in this unique and challenging population.

## Case presentation

**Figure 1:**
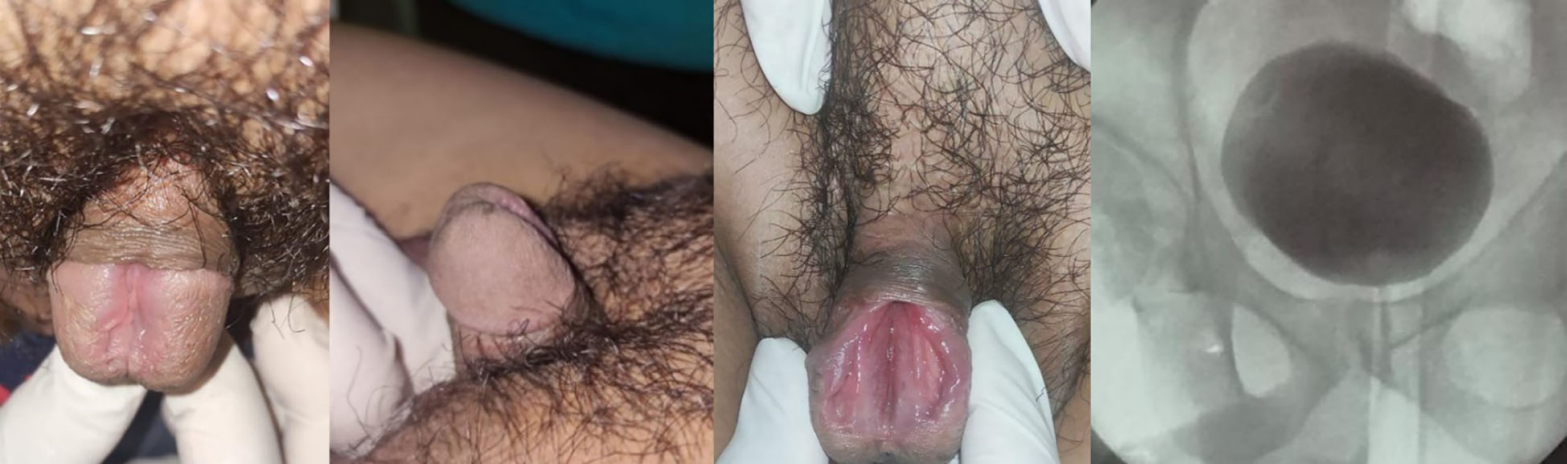
Preoperative clinical photographs showing dorsal urethral meatus at the midshaft and associated chordee in an adult male with isolated epispadias.

**Figure 2:**
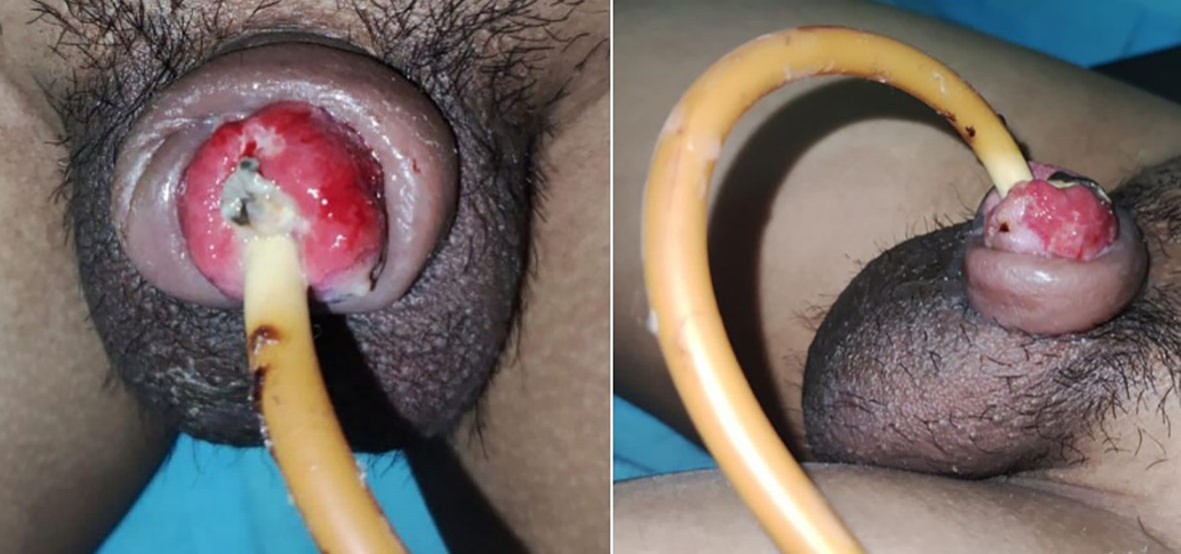
Postoperative clinical photographs demonstrating penile appearance and urethral meatus position after modified Cantwell-Ransley repair.

A 35-year-old man presented with untreated isolated epispadias. Although he was continent and exhibited no significant chordee, he elected to undergo surgical correction in anticipation of marriage. Physical examination revealed the urethral meatus at the midshaft of the dorsal penis, with a 4 cm wide longitudinal groove extending from the glans to the ectopic meatus (**Figure 1**). The testes and scrotal regions were unremarkable. Imaging demonstrated no pubic symphysis diastasis, and cystography confirmed the absence of vesicoureteral reflux. The patient underwent a modified Cantwell-Ransley procedure. After complete degloving, excision of fibrotic bands was performed to correct dorsal chordee, and the dorsal urethral plate was mobilized and transposed ventrally to construct a neourethra at the glans tip. The corpora cavernosa were approximated dorsally to provide stability, and the surgical wound was closed and dressed. On postoperative day 5, dressing removal revealed a mildly necrotic glans (**Figure 2**). The urinary catheter was removed on day 14, and the patient achieved normal urinary function without incontinence. At three months postoperatively, there were no complications such as fistula formation or wound dehiscence, and the patient reported satisfaction with both cosmetic and sexual outcomes. The clinical and surgical timeline of the case is summarized in **Table 1**.

**Table 1:** Timeline of care according to CARE guidelines.

Timeline	Even
Initial Visit	Diagnosis of adult isolated epispadias; marriage preparation.
Day 0	Modified Cantwell-Ransley procedure under general anesthesia.
Day 15	Identification of minor glans necrosis; started conservative treatment.
Day 30	Urinary catheter removal; normal voiding achieved.
3 Months	Follow-up: 16 ml/s, IIEF-5 score 22/25, high satisfaction.

### Diagnostic assessment

Physical examination revealed a urethral meatus at the midshaft of the dorsal penis, with a 4 cm wide longitudinal groove extending from the glans to the ectopic meatus (6, 7). Imaging using pelvic X-ray demonstrated no pubic symphysis diastasis, and cystography confirmed the absence of vesicoureteral reflux (8). Laboratory findings, including renal function and urinalysis, were within normal limits (9).

### Therapeutic intervention

The patient underwent a modified Cantwell-Ransley (MCR) procedure under general anesthesia, lasting approximately 150 minutes (10, 11). After complete degloving, excision of fibrotic bands was performed using PDS 6–0 and 5–0 sutures for the neourethra (5, 12). The MCR technique was preferred over Mitchell’s penile disassembly because it preserves the distal urethral plate’s attachment to the glans, minimizing the risk of extensive tissue ischemia in mature adult tissues (13, 14). The surgery followed the initial plan without intraoperative changes.

### Follow-up and outcomes

At the three-month follow-up, the patient reported high satisfaction with the cosmetic results (15). Objective measurements showed a of 16 ml/s via uroflowmetry and an IIEF-5 score of 22/25, indicating normal erectile function (11, 15). Minor glans necrosis observed on day 5 was managed conservatively with topical antibiotics and resolved spontaneously without tissue loss (5, 15).

## Discussion

The management of isolated epispadias in adults presents unique surgical challenges compared to the pediatric population. In adults, the penile tissues are less elastic, and the corpora cavernosa are fully developed, which can increase the risk of vascular compromise during extensive mobilization (11, 14). While the Mitchell technique (complete penile disassembly) offers excellent exposure, we opted for the Modified Cantwell-Ransley (MCR) procedure. The MCR technique allows for effective urethral mobilization and chordee correction while maintaining the distal attachment of the urethral plate to the glans, thereby preserving critical blood supply to the distal tissues (10, 12). This is particularly relevant in our case, where the transient glans ischemia remained superficial and resolved without permanent tissue loss, supporting the safety of MCR in mature tissues (5, 9).

Furthermore, the psychological burden in adult patients is often centered around sexual function and social integration, as seen in our patient who sought treatment specifically for marriage preparation (15). Unlike pediatric cases where the primary goal is achieving continence and anatomical normalcy, adult reconstructive urology must also prioritize erectile stability and subjective patient satisfaction (11). Our use of the IIEF-5 score and uroflowmetry provides objective evidence that functional success is achievable even in delayed presentations, bridging the gap between anatomical repair and quality of life (15).

### Patient perspective

The patient stated that the surgery significantly increased his self-confidence. He emphasized that achieving anatomical normalcy and normal sexual function was crucial for his psychological well-being and his readiness for marriage.

### Strengths and limitations of the approach

The strength of this study lies in the successful application of the MCR technique in a rare adult presentation with objective functional outcomes. However, this study is limited by its single-case nature, which restricts the generalizability of the findings. Additionally, the 3-month follow-up period is relatively short, and there is a potential for selection bias, as the patient was highly motivated by his upcoming marriage plans.

## Data Availability

The original contributions presented in the study are included in the article/supplementary material. Further inquiries can be directed to the corresponding authors.
